# Global gene expression analysis of early response to chemotherapy treatment in ovarian cancer spheroids

**DOI:** 10.1186/1471-2164-9-99

**Published:** 2008-02-26

**Authors:** Sylvain L'Espérance, Magdalena Bachvarova, Bernard Tetu, Anne-Marie Mes-Masson, Dimcho Bachvarov

**Affiliations:** 1 Department of Medicine, Laval University, Québec (Québec), Canada; 2 Department of Pathology, Laval University, Québec (Québec), Canada; 3 Cancer Research Centre, Hôpital L'Hotel-Dieu de Québec, Centre Hospitalier Universitaire de Québec (CHUQ), Québec (Québec), Canada; 4 Department of Medicine, Université de Montréal, Montreal, (Québec) Canada; 5 Centre de Recherche du Centre Hospitalier de l'Université de Montréal (CHUM), Institut du cancer de Montréal, Montréal (Québec), Canada

## Abstract

**Background:**

Chemotherapy (CT) resistance in ovarian cancer (OC) is broad and encompasses diverse unrelated drugs, suggesting more than one mechanism of resistance. To better understand the molecular mechanisms controlling the immediate response of OC cells to CT exposure, we have performed gene expression profiling in spheroid cultures derived from six OC cell lines (OVCAR3, SKOV3, TOV-112, TOV-21, OV-90 and TOV-155), following treatment with 10,0 μM cisplatin, 2,5 μM paclitaxel or 5,0 μM topotecan for 72 hours.

**Results:**

Exposure of OC spheroids to these CT drugs resulted in differential expression of genes associated with cell growth and proliferation, cellular assembly and organization, cell death, cell cycle control and cell signaling. Genes, functionally involved in DNA repair, DNA replication and cell cycle arrest were mostly overexpressed, while genes implicated in metabolism (especially lipid metabolism), signal transduction, immune and inflammatory response, transport, transcription regulation and protein biosynthesis, were commonly suppressed following all treatments. Cisplatin and topotecan treatments triggered similar alterations in gene and pathway expression patterns, while paclitaxel action was mainly associated with induction of genes and pathways linked to cellular assembly and organization (including numerous tubulin genes), cell death and protein synthesis. The microarray data were further confirmed by pathway and network analyses.

**Conclusion:**

Most alterations in gene expression were directly related to mechanisms of the cytotoxics actions in OC spheroids. However, the induction of genes linked to mechanisms of DNA replication and repair in cisplatin- and topotecan-treated OC spheroids could be associated with immediate adaptive response to treatment. Similarly, overexpression of different tubulin genes upon exposure to paclitaxel could represent an early compensatory effect to this drug action. Finally, multicellular growth conditions that are known to alter gene expression (including cell adhesion and cytoskeleton organization), could substantially contribute in reducing the initial effectiveness of CT drugs in OC spheroids. Results described in this study underscore the potential of the microarray technology for unraveling the complex mechanisms of CT drugs actions in OC spheroids and early cellular response to treatment.

## Background

Ovarian cancer (OC) is the fourth commonest cause of cancer related death in women [1]. The majority of patients present with advanced disease, with an overall five-year survival rate of approximately 30–40% following debulking surgery, initial platinum-based CT and further CT at relapse [1]. Combination CT with paclitaxel and a platinum compound (carboplatin or cisplatin) is the current regimen of choice for the treatment of advanced OC [2]. A number of clinical issues, however, are unresolved including drug dosage and schedule, duration of treatment, and route of administration [2]. Thus, although significant proportions of women respond to CT, the majority of responders (approximately 50%–75%) eventually relapse at a median of 18 to 28 months [3]. Treatment decisions at this juncture include supplementary CT with topotecan, hormones, surgery, and experimental agents [4]. Nonetheless, even with these additional treatments, relapse rates remain high and most women with advanced OC ultimately will die of their disease [5]. CT resistance in OC is broad and encompasses diverse unrelated drugs, suggesting more than one mechanism of resistance. A number of other cellular factors have increased expression and activity in drug-resistant OC cell lines and/or tumor tissues [reviewed in [6]]. However, for the majority of these factors, *in vivo *studies have failed to assess their clinical importance and to translate them into recommendations for specific therapies or prognosis in OC patients [7].

The recent advent of microarray-based profiling technologies has provided an opportunity to simultaneously examine the relationship between thousands of genes and clinical phenotypes. Using this approach, several groups, including ours, have tried to identify gene expression signatures and/or specific biomarker sets of response to first-line platinum-based CT in OC following debulking surgery [8-13]. These studies have identified different prognostic and predictor gene sets which can distinguish early from late relapse or disease progression; however, no significant overlap was found between the individual predictor lists. Recently, we used an alternative approach to evaluate the global gene expression in paired tumor samples taken prior to and post CT treatment from six patients with predominantly advanced stage, high-grade OC [14]. We have identified a number of genes that were differentially expressed in post-CT tumor samples, including different factors associated with tumor invasion/progression, control of cell proliferation, and chemoresistance. However this approach could not reveal mechanisms of early response to CT treatment since post-CT OC tumors were available 3 to 40 months following the last CT treatment [14].

In this study, we have chosen the versatile multicellular spheroid model [15] to assess early drug action and instant response to CT treatment in OC cells. Indeed, experimental three-dimensional models such as multicellular spheroids may provide a better *in vitro *approximation of solid tumors [15], and have been used for study of multicellular resistance [16,17]. Since the rapid acquisition of resistance probably represents a physiologic mechanism of adaptation at the multicellular level and not a stable genetic change [18], the spheroid model seems to be more appropriate to study early occurrence of acquired drug resistance in solid tumors than monolayer cell cultures [15]. Thus, monolayers do not pose the barrier to drug penetration or provide many of the microenvironmental influences found in solid tumors and 3D cultures [16]. Experimental data indicating that initial exposure to drug *in vivo *may induce low, but yet clinically significant transient resistance [17-19], also support this hypothesis. Herein, we applied the DNA microarray technology to investigate the cellular and molecular mechanisms implicated in immediate drug action and early cellular compensatory response to drugs, commonly used as first- or second-line treatment of OC, including cisplatin, paclitaxel and topotecan. We present evidence that initial defense reactions in OC spheroids are mostly associated with the induction of DNA repair pathways and the implication of multicellular/adhesion-dependent or -associated mechanisms.

## Results

### Common gene expression signatures of OC spheroids following treatment with all drugs used (cisplatin, paclitaxel and topotecan)

We employed Agilent Human oligonucleotide microarrays, containing ~22,000 genes to identify global gene expression changes in spheroids propagated from six different OC cell lines (OVCAR-3, SKOV-3, OV-90, TOV-21, TOV-112, TOV-155), following treatment with three different CT drugs (cisplatin [10 μM], topotecan [5 μM] and paclitaxel [2.5 μM]) for 72 hours. For each drug, the concentration used was empirically estimated as the maximal drug concentration which does not cause a considerable cell death (less than 20%) and/or changes in spheroid morphology during the treatment period (data not shown). Those concentrations were significantly higher than the IC50 values determined for each cell line, when grown as monolayer (Table 1).

**Table 1:** Characteristics of the six OC cell lines used in the study.

Cell type	Source	Histopathology	IC50 values (μM)		
			
			cisplatin	paclitaxel	topotecan
OVCAR-3	tumor	Adenocarcinoma	0,70	0,03	0,27
SKOV-3	ascites	Adenocarcinoma	7,50	0,05	0,23
OV-90	ascites	Adenocarcinoma	4,50	0,025	0,21
TOV-21	tumor	Clear cell carcinoma	2,00	0,013	1,3
TOV-112	tumor	Endometrioid carcinoma	3,50	0,016	0,25
TOV-155	tissue	Cyst adenoma^a^	1,25	0,015	0,27
Treatment^b^			10,0 μM	2.5 μM	5,0 μM

^a^Upon cultivation, the spontaneously immortalized TOV-155 cell line exhibited typical characteristics of an OC cell line (including p53 mutations, oncogene expression and continuous growth). The complete characterization of this cell line will be reported elsewhere.

^b^The drug concentrations indicated were used for treatment of the OC spheroid cultures (details in text).

First, we compared shared gene expression patterns between control (non-treated) and all cisplatin-, topotecan- and paclitaxel-treated spheroids derived from the six cell lines studied, in search for common markers and/or molecular mechanisms involved in drug action and immediate treatment response. A subset of 971 differentially expressed genes was selected from all microarray data by initial filtering on confidence at *p*-value = 0.05, followed by filtering on expression level (≥1.5 fold). Using these selection criteria, we found 348 genes to be commonly up-regulated and 623 genes to be down-regulated in the CT drugs-treated spheroids [see Additional file 1]. Table 2 shows a list of selected functionally related groups of genes that were differentially expressed (≥1.5-fold) in all treated spheroids. As seen from Table 2, comparable numbers of genes with previously shown implication in mechanisms of apoptosis, cell adhesion, cell cycle control, and stress (defense) response were both up- and down-regulated in the treated OC spheroids. Genes, functionally associated with DNA repair, DNA replication and cell cycle arrest were exclusively overexpressed (Table 2A). Genes implicated in cell growth and maintenance, transcription regulation, signal transduction and transport were predominantly down-regulated, while genes linked to immune and inflammatory response, metabolism (especially lipid metabolism), protein biosynthesis, protein modification and RNA processing, were uniquely suppressed following all treatments (Table 2B).

**Table 2:** Selected common differentially expressed gene groups upon treatment with all drugs (cisplatin, topotecan and paclitaxel).

**A. Up-regulated genes**	
Apoptosis	*BMF, CASP3, CASP7, EMP1, FADD, FOSL2, NOL3, PDCD2L, PERP, PHLDA2, ZNF443, BIRC3, BCL2A1*
Cell adhesion	*BAIAP1, CELSR3, CLDN2, CLDND1, COL15A1, COL17A1, COL8A1, CSPG3, GJA10, OIP5, SSX2IP, SYMPK, TNXB*
Cell growth and maintenance	*CDCA7, DCC1, KNTC2, NRG4, PRC1, SPATA5L1, TGFA, NOV*
Cell cycle	*CNNM4, BUB1, CCNB2, CDC20B, CDCA2, CKS2, KIF23, PKMYT1, CDCA5, FLJ23311*
Cytoskeleton	*ANXA9, C16orf5, CKAP2, K-ALPHA-1, KIAA1524, KIF18A, PALMD, SDCBP, SPTAN1, TUBB2, TUBB3, TUBB6*
DNA replication and repair	*BRCA1, BRCA2, DDB2, FANCA, MCM10, MCM8, NP, PCNA, PTTG1, RPA3, POLA2, RRM1*
Cell cycle arrest	*BTG3, CDKN1A, CDKN2D, GMNN, HIS1, RFP2*
Regulation of transcription	*DAZL, DXYS155E, ELL2, HPS3, KLF5, LHX2, NR4A1, PER2, PHTF1, POLR2A, RFXAP, ST18, TAL2, TRIP13, ZNF211, ZNF304, ZNF326, ZNF483, ZNF529, ZNF541, ZNF550, ZNF555, ZNF646, ZNF701, ZIC4*
Response to stress	*AHR, HSFY2, HSPH1, MICB, TXNDC, YWHAB*
Intracellular signaling (signal transduction)	*ASB3, AURKC, CALM2, CXXC4, DBF4B, DGKG, DKK1, DUSP8, DUSP9, EFNA3, FZD4, GPR109B, GPR6, IMP-1, MIP, MS4A3, NMI, NRAS, NYD-SP25, OR5P2, OR6K2, OR9I1, PDC, RAB21, RAB2B, RACGAP1, RGS13, RKHD3, SH2D1A, STMN1, STRN4, TAS2R60, WNT10A*
Transport	*ABCC9, AQP11, ATP2B3, CACNB1, CHAC2, KCNJ9, MGC29671, NUP107, Shax3, SLC19A2, SLC25A17, SLC7A8, TRPM6, TXNL5, RAMP*

**B. Down-regulated genes**	

Apoptosis	*AIFM3, BCLAF1, DAPK3, DOCK1, FAF1, FKSG2, GADD45B, HIPK2, NALP12, NTN1, SPOCK, TNFRSF6B, YARS*
Cell adhesion	*CASK, CD151, CIB3, CLDN18, CLDN5, CNTN4, GJC1, ITGB1, ITGB3BP, ITM2C, LAMA5, LOC388419, PKP4, PSTPIP1, PTK7, TNR*
Cell growth and maintenance	*TBC1D5, CDC2L5, HDGF2, AKIP, AAMP, LRPAP1, MATK, PDAP1, PPARGC1B, SIPA1L3, TPD52L2, MT3, POLDIP2, POLDIP3, SET, ASCC3*
Cytoskeleton	*ACTR1B, ARPC1A, ARPC4, CKAP1, CORO1B, DNAI2, FMNL1, KRT6C, PALM2, SPAG7, SPTBN5, TNK2, TUBGCP2, VILL, YWHAG*
Response to stress	*CAMP, CEBPE, DEFB1, DEFQ1, GATA3, HSPA1A, HSPA12B, NOD27*
Immune & inflammatory response	*ALCAM, AMBP, C1QB, C1QL1, C5, CD2BP2, COLEC11, HLA-A, HLA-B, HLA-E, HLA-F, IFI35, IGSF4, IL16, KIR3DL3, LAT, OAS3, R30953_1, RFX1, SAA2, TNFRSF13B, TREM1, CARD10, CCL19, EDARADD, LGALS9, SN*
Lipid metabolism	*ACAA1, AKR1C3, APOE, ARH, ASAH1, CHKA, GPX4, HDLBP, HMGCS1, INPP5E, LDLR, MGLL, PCCB, PEMT, PIP5K2A, PLA2G12B, PLCB3, PRKAG1, PTE1*
Metabolism (other than lipid metabolism)	*ADH5, AK5, AKR1C1, ALDH4A1, ALDOA, ASMTL, ATAD4, B4GALT2, BCDO2, CA5A, CHST7, CYP11A1, DHPS, DHRS10, DOT1L, DPYD, ENO1, ENO1B, FBXW5, GAL3ST3, GMDS, GRHPR, HAAO, HCG9, HK2, HPD, IMPDH2, INSR, ISOC2, ITGB1BP2, ITIH2, LARGE, LEPREL2, MAT2A, MPPED1, MSRA, MVK, NAT6, NNMT, NUDT14, PGAM5, PHGDHL1, PKLR, PTPRG, PYGM, RDH13, SDHA, TKT*
Protein biosynthesis & modification	*C19orf28, CRYL1, EEF1A2, EEF1D, EEF1G, EIF3S5, EIF3S8, EIF4B, EIF4G1, EIF5A, HAGHL, KRT6E, MRPL12, MRPL23, RPL10L, RPL6, RPS14, RPS19, RPS2, RPS9, VARS2, ANKRD13D, ATAD3A, ATAD3B, AFG3L2, ADCK1, ADCY5, BCKDK, CRYAA, DUSP15, FKBP10, FLOT1, GALNT9, HKE2, KIAA1542, MAP2K2, MTMR4, PARP10, PCTK2, PPP2R4, PPIB, SERPINH1, SIL1, SRPK2, SSTK, ST3GAL4, WNK2*
Cell cycle	*CCND1, DLG1, DNM2, DDIT3, EXT1, MAD1L1, PLK1, PPP1R9B, SEPT2, STAG1*
Regulation of transcription	*ARID3A, ASCL2, ATF4, CIRBP, CITED4, CORO1A, DBP, DEAF1, EWSR1, FOXQ1, GSCL, HES6, HMG20B, ING5, IPF1, KLF13, LBX1, MAF1, MBD3, MED25, MED31, MLLT1, NKX2-8, P4HB, PITPNM1, POLR1A, POLR2E, PRDM9, SEC8L1, SOX4, TBL1XR1, TCF25, TEAD2, TGFB1I4, VGLL4, ZFP276, ZFPM1, ZNF214, ZNF225, ZNF425, ZNF511, ZNF569, ZNF768, DHCR24, MXD4, TUFM*
RNA processing	*RALY, SURF6, ARL6IP4, CPSF3L, DDX56, HNRPA2B1, HNRPAB, LOC144983, PABPC4, PARN, PSD, RPP40, SFRS5, SNRPB, SNRPN, U1SNRNPBP*
Intracellular signaling (signal transduction)	*AKT2, ANKRD23, ARHGEF15, ARL2, BRE, CABIN1, CKB, CSF3, CSNK1A1L, DHH, FZD1, GNA15, GNAQ, GNB2L1, GPR172B, GPR31, GPR32, GPR37L1, GPS1, GRAP, GRK1, GRM4, HRMT1L2, IGF2R, IL17R, INADL, JAK1, KSR2, MAP4K5, MAPK12, MAPK13, MAPKAPK2, MRAS, NMBR, NRTN, NSMCE1, NXF, OMP, OPN1LW, OPRD1, OR2T5, OR2Z1, OR8G5, OXT, PDHA2, PIK3R2, PIK4CA, PITPNC1, PKIG, PRKCSH, PTK9L, PTPN11, PTPRM, RAB3D, RHO, RHOC, RHPN1, RSU1, SRGAP3, STK25, PAC1BP3, TMEPAI, TNNT3, TRIM54, USH1C, VRK3, WNT3A*
Transport	*ABCC4, ABCC6, ARL7, ATP1A3, ATP6V0E2, CACNG4, CHMP6, COL4A1, COX7A2L, CYBA, CYC1, ETFDH, EXOSC6, FMNL2, GABARAP, GRPEL1, HBM, HOXB8, ITPR2, ITPR3, KCNJ13, LMAN2, MGC19604, MLPH, MSCP, MYBBP1A, NBEA, NDUFS2, NDUFS6, PEX26, RAB1A, RALB, RAP2B, SCNN1D, SEC14L5, SEC24C, SEC61A1, SFXN4, SGNE1, SLC12A6, SLC17A6, SLC1A5, SLC25A24, SLC25A29, SLC25A6, SLC37A1, SLC6A8, SNX15, SNX17, SORT1, STX10, TIMM44, TLOC1, TMED9, TOMM40, TRAPPC6A, TRIAD3, TRPC5, TXN2, TXNRD1*

**Figure 1:**
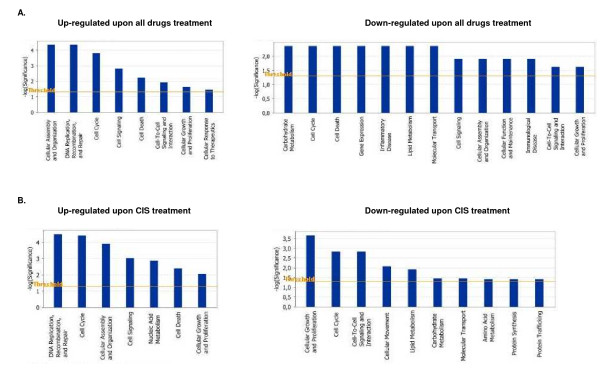
Functional analysis for a dataset of differentially expressed genes (≥1.5 fold) in OC spheroids following CT drugs treatments. A. Functional analysis following all drugs (cisplatin, topotecan and paclitaxel) treatment, B. Functional analysis following cisplatin treatment. Top functions that meet a *p*-value cutoff of 0.05 are displayed.

Pathway and network analyses based on the 971 gene list were generated through the use of Ingenuity Pathways Analysis (IPA). The IPA analysis confirmed the major functionally related groups, found to be commonly up- or down-regulated in drugs-treated OC spheroids. Thus, pathways linked to cell growth and proliferation, cellular assembly and organization, cell death, cell cycle control and cell signaling were both induced and suppressed; pathways functionally related to DNA replication, recombination and repair and cellular response to therapeutics were induced, while pathways associated with control of gene expression, metabolism, transport, immune and inflammatory response displayed suppression upon treatments with all drugs (Figure 1A).

**Figure 2:**
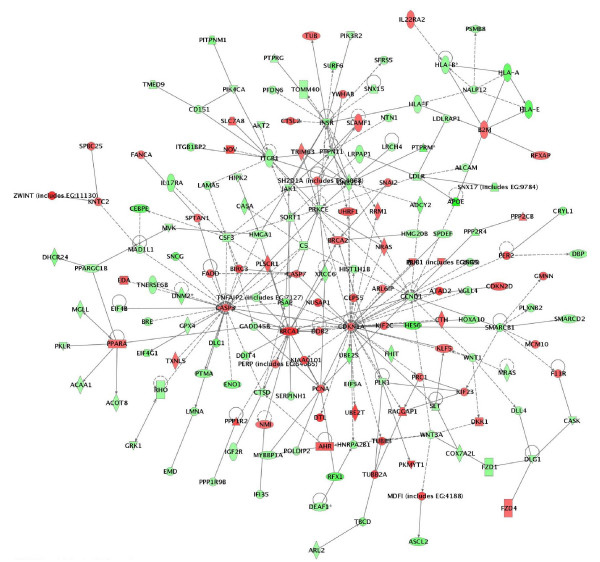
Network analysis of dynamic gene expression in OC spheroids based on the 1.5-fold common gene expression list obtained following treatment with all CT drugs used (cisplatin, topotecan and paclitaxel). The five top-scoring networks were merged and are displayed graphically as node (genes/gene product) and edges (the biological relationships between the nodes). Intensity of the node color indicates the degree of up- (red) or downregulation (green). Nodes are displayed using various shapes that represent the functional class of the gene product (square, cytokine, vertical oval, transmembrane receptor, rectangle, nuclear receptor, diamond, enzyme, rhomboid, transporter, hexagon, translation factor, horizontal oval, transcription factor, circle, other). Edges are displayed with various labels that describe the nature of relationship between the nodes: ---- binding only, → acts on. The length of an edge reflect the evidence supporting that node-to-node relationship, in that edges supported by article from literature are shorter. Dotted edges represent indirect interaction.

A network analysis identified 34 highly significant networks with score ≥ 13 [see Additional file 2]. As expected, the five top-scoring networks were associated with functions linked to cellular growth and proliferation, cell cycle, cell death, cellular movement and metabolism (Table 3A). A common network obtained upon merging the five top-scoring networks (Figure 2) recognized several important nodes linked with numerous interaction partners, including cyclin-dependent kinase inhibitor 1A (*CDKN1A*, *p21*, *Cip1*), caspase 3 (*CASP3*), breast cancer 1 (*BRCA1*), proliferating cell nuclear antigen (*PCNA*), peroxisome proliferative activated receptor alpha (*PPARA*), cyclin D1 (*CCND1*), insulin receptor (*INSR*), integrin beta 1 (*ITGB1*), guanine nucleotide binding protein beta polypeptide 2-like 1 (*GNB2L1*), protein kinase C epsilon (*PRKCE*), SWI/SNF related, matrix associated actin dependent regulator of chromatin subfamily b, member 1 (*SMARCB1*), protein tyrosine phosphatase, non-receptor type 11 (*PTPN11*), low density lipoprotein receptor (*LDLR*), major histocompatibility complex class IA (*HLA-A*), discs, large homolog 1 (Drosophila) (*DLG1*), polo-like kinase 1 (*PLK1*) and colony stimulating factor 3 (*CSF3*). While the up-regulated gene nodes and related pathways were mostly associated with cell cycle arrest, induction of apoptosis (*CASP3*, *CDKN1A*) and DNA repair (*BRCA1*, *PCNA*), the down-regulated gene nodes were predominantly linked to control of cell cycle progression and cell proliferation (*CCND1*,*GNB2L1*,*SMARCB1*,*DLG1*,*PLK1*), carbohydrate and lipid metabolism (*INSR*, *LDLR*), intracellular signaling (*PRKCE*, *PTPN11*), cell adhesion (*ITGB1*) and immune response (*HLA-A*, *CSF3*). *PPARA *up-regulation could also contribute to reduced metabolism rates in treated spheroids by negatively regulating genes implicated in carbohydrate (*PKLR*) and mostly lipid (*ACOT8*, *ACAA1*, *MGLL*, *PPARGC1B*, *DHCR24*) metabolism (Figure 2).

**Table 3:** Genetic networks in CT drugs-treated OC spheroids^a^.

Ntwk	Genes in Ingenuity networks^b^	Associated pathways	Score^c^
*A. Networks, commonly affected by treatment with all drugs (cisplatin, topotecan and paclitaxel)*			

1	***ARL2, ARL6IP, ATAD2, BUB1 (includes EG:699), CDKN1A, CEP55, CTH, DEAF1, DTL, EIF5A, FHIT, HES6, HIST1H1B, HNRPA2B1, HOXA10, ING5, KIAA0101, KIF23, KIF2C, NUSAP1, PBK, PKMYT1, PLK1, PRC1, PSAP, RACGAP1, SORT1, SPDEF, TBCD, TUBB3, TUBB2A, UBE2S, UBE2T, UHRF1, VGLL4***	Cell Cycle, Cellular Movement, Cancer	43
2	***BIRC3, BRE, CASP3, CASP7, CEBPE, CSF3, CTSD, DLC1, DNM2, EDA, EIF4B, EIF4G1, EMD, ENO1, FADD, FANCA, GPX4, GRK1, IGF2R, IL17RA, KNTC2, LAMA5, LMNA, MAD1L1, PLSCR1, PRKCE, PTMA, RHO, SNCG, SPBC25, SPTAN1, TNFRSF6B, TRIM63, TXNL5, ZWINT***	Cell Death, Connective Tissue Disorders, Cellular Growth and Proliferation	43
3	***ADCY2, AHR, ALCAM, APOE, ASCL2, CASK, CCND1, CDKN2D, COX7A2L, CRYL1, DBP, DKK1, DLG1, DLL4, F11R, FZD1, FZD4, GMNN, KLF5, LDLR, LDLRAP1, LRPAP1, MCM10, MDFI (includes EG:4188), MRAS, PER2, PLXNB2, PPP2CB, PPP2R4, SET, SMARCB1, SMARCD2, SNX17 (includes EG:9784), WNT1, WNT3A***	Gene Expression, Lipid Metabolism, Small Molecule Biochemistry	43
4	***AKT2, BRCA1, BRCA2, C5, CA5A, CD151, DDB2, DDIT4, GNB2L1, HMG20B, IFI35, ITGB1, ITGB1BP2, JAK1, LRCH4, NMI, NOV, NRAS, NTN1, PERP (includes EG:64065), PIK4CA, PITPNM1, PPP1R2, PPP1R9B, PTPN11, PTPRM, SERPINH1, SH2D1A (includes EG:4068), SLAMF1, SLC7A8, SNAI2, TMED9, TNFAIP2 (includes EG:7127), XRCC6, YWHAB***	Cell Death, Cancer, Cell Cycle	43
5	***ACAA1, ACOT8, B2M, CTSL2, DHCR24, GADD45B, HIPK2, HLA-A, HLA-B, HLA-E, HLA-F, HMGA1, IL22RA2, INSR, MGLL, MVK, MYBBP1A, NALP12, PCNA, PFDN6, PIK3R2, PKLR, POLDIP2, PPARA, PPARGC1B, PSMB8, PTPRG, RFX1, RFXAP, RRM1, SFRS5, SNX15, SURF6, TOMM40, TUB***	Nutritional Disease, Cancer, Lipid Metabolism	43

*B. Networks, affected by cisplatin treatment*			

1	***BRCA1, CEBPE, CLU, DDIT4, ECE1, EEF1D, ENO1, EPPB9, GATA3, HIPK2, LDLR, LDLRAP1, LTBP3, MAD1L1, MAPKAPK2, MGMT, MSH2, MXD4, PDZK1IP1, PERP (includes EG:64065), PKIG, PLSCR1, PRKCG, PSMC3, PSMD12, RAG2, RHO, SMAD3, SPHK1, STOML2, TGFB1, TGIF, TMEPAI, TNFAIP2 (includes EG:7127), XRCC6***	Cell Cycle, Cancer	51
2	***ATAD2, CDC2, CDKN1A, CHAF1B, DKFZP762E1312, DNMT1, DTL, EXO1, FHIT, HES6, HOXA10, ING5, KIAA0101, MCM2, MCM3, MCM4, MCM10, MYBBP1A, NUSAP1, PBK, PCNA, PIP5K1C, PKMYT1, PSAP, RACGAP1, RRM1, RRM2, RUVBL2, TYMS, UBE2A (includes EG:7319), UBE2S, UBE2T, UHRF1, VGLL4, WDHD1***	DNA Replication, Recombination, and Repair, Cancer	51
3	***APAF1***, *APIP, AVEN*, ***BCL2A1, BDNF, BIN1***, *BIRC1*, ***CASP3, CASP7***, *CSH2*, ***CTSD***, *DBNL, DEDD, DFFA*, ***DLC1***, ***DNM2***, ***EIF4G1***, *EIF4G3*, ***FADD***, *GAS2*, ***GPX4***, *HCRT, HSH2D*, ***ITGB3BP, LTBR, MAP2K5***, *PDE4A, PINK1, PRKAA2*, ***PRKCZ, PSAP, SNCG, STK11, TGFA, TNFRSF6B***	Cell Death, Cancer, Hematological Disease	21
4	*BRD2, C19ORF2, CREB1*, ***CYP11A1***, *EP300*, ***EWSR1, EXOSC6***, *FUSIP1*, ***GAS2L1, HLA-G, HSPA4L, ITM2C***, *LSM7*, ***MYH13***, *MYOD1, NFYB*, ***NUDT2, POLR1A***, *POLR2A*, ***POLR2E***, *POLR2F*, ***RRN3, SENP1, SIAHBP1, SUPT3H***, *TAF1C, TBP*, ***TEAD2, TEAD4, TKT***, *TNNC1*, ***TNNI2, TNNT2***, *TRERF1, VGLL1*	Gene Expression, Cell Cycle, Skeletal and Muscular System Development and Function	20
5	***ALAS1***, *ALPP, ARPC5, CD244*, ***CD2BP2***, *CTH, CUGBP2*, ***DNAJB5, EDF1***, *EGR2*, ***EPS8L2***, *ERG, FOS*, ***GRM4, HDLBP, HLA-B***, *HOXA9, IL1RL1*, ***JUB***, *JUN*, ***KEAP1***, *NFE2L2*, ***NIPSNAP1***, *NTS (includes EG:57303)*, ***PMP22, RPS9, SDCBP, SKIV2L, SNCG, SNRPB***, *SOS1*, ***USP1***, *WBP4*, ***WDR90***, *WT1*	Gene Expression, Organismal Injury and Abnormalities	18

*C. Networks, affected by topotecan treatment*			

1	***ARL6IP, ASPM, ATAD2, CDKN1A, COG1, COG5, CRI2, DDB2, DEAF1, DLG7, FHIT, H2AFZ, HES6, HIST1H1B, HNRPA2B1, KIF2C, KLF5, LIG3, MLLT1, NEIL1, NUSAP1, PARP2, PLK2, PSAP, RACGAP1, SET, SPDEF, SUPT16H, TRIM44 (includes EG:54765), UBE2C, UBE2D1, UBE2S, UBE2T, UHRF1, VGLL4***	DNA Replication, Recombination, and Repair, Cell Cycle, Cellular Compromise	41
2	***ADCY2, ADCY5, AKT1, ASCL2, CCL19, CCND1, CDH2, DKK1, DLC1, DLG1, DLG2, DLL4, FZD1, FZD4, GNAI1, GNB1, GNB2L1, H2AFX, MDFI (includes EG:4188), MRAS, NEUROG1, NEUROG3, NRAS, PRKAG1, PRKAR1A, PTPRM, RASSF1, ROBO1, SEMA3C, SRGAP1, TBCD, TUBB3, TUBB2A, WNT1, WNT3A***	Cellular Movement, Cancer, Reproductive System Disease	41
3	***AMPH, BRE, CABIN1, CALM1, CASP3, DAPK1, DDEF1, DNM2, DOCK1, EIF4B, EIF4G1, EIF4G3, ENO1, EPN1, EPS15, GNLY, GRK1, LMNA, MAP4K3, PTK2, PTMA, RHO, RIT2, SNCG, SPHK1, SPTAN1, SPTBN1, STK3, SYNE2, TNFRSF6B, TPD52L1, TPD52L2, TRIO, TXNL5, WASF1***	Cellular Assembly and Organization, Cellular Function and Maintenance, Cellular Movement	41
4	***ALDOA, ANP32B, B2M, BRF1, CALR, CAMK1, CKB, CLIC4, CORO1A, ELAVL1, FN1, GABARAP, HLA-A, HLA-B, HLA-E, IGF1R, ITGB5, ITGB3BP, ITSN1, KPNA2, KRT8, MAPK7, MEF2A, NALP12, NTN1, NUMB, PTPN11, PTPN3 (includes EG:5774), RAB1A, RAN, SLC2A4RG, SND1 (includes EG:27044), VCL, YWHAB, ZAK***	Protein Trafficking, Cell Death, Molecular Transport	41
5	***ACTL6A, ACTL6B, ARID1B (includes EG:57492), CCNB2, CDC2, CDK8, CEP170, CGA, CTSL2, GMNN, HMGA2, IFITM2, IFNGR1, INSR, ISGF3G, JAK1, MED25, NEK2, PKMYT1, PLK1, PLXNB2, PRKCZ, PSMA4, PSMB7, PTPRG, PTTG1, RAD51AP1, RNF103, SFRS5, SLC9A3R1, SMARCB1, SMARCD2, SNX15, SURF6, TUB***	Cell Cycle, Cellular Assembly and Organization, Cancer	41

*D. Networks, affected by paclitaxel treatment*			

1	***BAG5***, *CTNNB1, CTNNBIP1, FZD8, GSTP1 (includes EG:2950), H2-ALPHA, JRK*, ***K-ALPHA-1***, *KIF23*, ***KLK2***, *LOC112714*, ***LRP6, MDFI (includes EG:4188), MRPL13, MT3***, *MYF5, PARK2*, ***PLK1, PTP4A3, RBP4***, *SERPINA5, TPT1, TUBA1*, ***TUBA2, TUBA3, TUBA6***, *TUBA8*, ***TUBB***, *TUBB1*, ***TUBB3, TUBB4, TUBB2A***, *TUBB2C, TUBG1, WNT3A*	Cancer, Reproductive System Disease, Renal and Urological Disease	22
2	*ALOX5AP*, ***ASAH2, CEBPE***, *CEBPG*, ***DDIT3, DHPS, DNAJB5, DUSP9 (includes EG:1852), EIF4G1***, *ELK4, ERN1 (includes EG:2081)*, ***G6PD***, *GCLC, GCLM, IL3*, ***IMPDH2, LAT***, *LCN2*, ***MAPK12***, *MKNK1, MKNK2, MMP8*, ***MT2A***, *NFE2L2, PRG2 (includes EG:5553), RPS6KA5 (includes EG:9252), SLC5A5, SLK, TALDO1*, ***TDRD7, TGIF***, *TLR9, TNF*, ***WNT10A***, *ZBTB17*	Drug Metabolism, Molecular Transport, Small Molecule Biochemistry	19
3	***ARPC1A***, *ARPC1B*, ***BAX***, *BIRC6, CCNL2*, ***CXCL13, DDIT4***, *EIF5A, FFAR3*, ***GATA3***, *GPR44*, ***HLA-A, HLA-E, HNRPA2B1, HOXC11***, *HSD17B1*, ***ING5***, *JMY, LETMD1*, ***LMNA, LTB***, *LTBP1*, ***MDH1***, *MDM4, NALP12, PEG3 (includes EG:5178), PPP1R13B, PYCARD*, ***RALY***, *SERPINB5, STK11*, ***TBX21***, *TP53, TP53INP1, VHL*	Cancer, Cell Death, Skeletal and Muscular Disorders	19
4	***AHNAK, ANP32B***, *BAK1, C19ORF10, CAMP*, ***DBP, DDIT3***, *ELAVL1*, ***ENO2***, *FCGR2A, FCGR2B, HLA-C*, ***HNRPA2B1***, *HRAS, IFI202B, IFNG, IL6, IL24, IL1RL1*, ***LGALS1, LY6E***, *MOG, PLCE1, PSMB9, PSMB10*, ***PTMA, S100A10, SDHA***, *SIRPA, TAP1*, ***TFG***, *TNFRSF10B, TNFRSF6B*, ***TRIM21, TTC28***	Connective Tissue Disorders, Inflammatory Disease, Cell Death	17
5	*ATF6*, ***ATP5B, BZRAP1***, *COL1A1, COX4I2, COX5B, COX6A1*, ***COX6A2***, *COX6B1, COX6B2, COX7A2L, COX7B, COX8C*, ***CREBL1***, *DBI, GATAD2B, HDAC1 (includes EG:3065)*, ***ITM2C***, *LY6A*, ***MBD3 (includes EG:53615)***, *MBD3L1*, ***MRPL12***, *MT1A, MYC, NFYB*, ***PLK1, PRRG2, RAB3D***, *RIMS1, RIMS2*, ***TRAM2, TSPO***, *TXNIP*, ***UBE2S, ZBTB16***	Cancer, Gene Expression, Tumor Morphology	17

^a^The five top-scoring networks for each drug treatment are presented.

^b^Bold genes are those identified by the microarray analysis, other genes were either not on the expression array or excluded by our gene expression selection criteria.

^c^A score of 3 was considered significant (p < 0.001).

### Specific changes in spheroids gene expression following treatment with cisplatin, topotecan or paclitaxel

In parallel, we separately analyzed the gene expression profiles in all six spheroid cultures following treatments with each of the three drugs used (cisplatin, topotecan or paclitaxel). The gene expression was compared between control and treated spheroids, and a subset of differentially expressed genes was selected displaying at least 1.5-fold difference in four of the six microarray experiments performed for each drug treatment.

#### Cisplatin treatment

Using these selection criteria, we found 205 genes to be up-regulated and 363 genes to be down-regulated in OC spheroids following cisplatin exposure [see Additional file 3]. Table 4 shows list of selected functional groups of genes that were differentially expressed (≥1.5-fold) in the cisplatin-treated spheroids. As seen from Table 4, up-regulated major functional gene groups comprised genes mostly involved in cell growth and proliferation, control of cell cycle (including cell cycle arrest) and DNA replication and repair. Down-regulated genes upon cisplatin treatment were functionally associated with immune and inflammatory response, metabolism, protein biosynthesis and modification, RNA processing, signal transduction and transport. Genes involved in cell adhesion, chromatin organization, cytoskeleton structure and regulation of transcription were predominantly suppressed following cisplatin exposure. Apoptosis genes were proportionally up- and down-regulated.

**Table 4:** Selected differentially expressed gene groups upon cisplatin treatment.

**A. Up-regulated genes**	
Apoptosis	*DNAJA3, EMP1, PERP, PHLDA2, ZNF443, BCL2A1, APAF1, CASP3, CASP7, FADD, PPP2CA*
Cell growth and proliferation	*DSN1, MIS12, MND1, SPATA5L1, DCC1, HELLS, ANXA1, NUSAP1, Spc25, TGFA, TYMS, UHRF1, EZH2*
DNA replication and repair	*BRCA1, CHAF1B, EXO1, MSH2, NEIL3, PCNA, POLQ, RAD51AP1, UBE2A, PRIM2A, MCM10, MCM2, MCM3, MCM4, POLA2, POLE3, PRIM1, RRM1, RRM2*
Cell cycle	*ZWINT, SEPT10, CDC2, CDC45L, CDCA5, CNNM4, E2F8, GNL3, PKMYT1, PPP6C*
Cell cycle arrest	*BTG3, CDKN1A, GMNN, HIS1, RECK, CDKN2D*
Cell adhesion	*CLDN2, TNXB, GJA10, EMP1, ANKRD32*
Cytoskeleton	*PALMD, SDCBP, ANKRD27*
Regulation of transcription	*C16orf34, CBFB, CREM, ELL2, MORC3, NR4A1, PHTF1, RRN3, TRIP13, WDHD1, ZBTB38, ZNF180, ZNF211, ZNF26, ZNF304, ZNF326, ZNF45, ZNF529, ZNF550, DNMT1, EED, TGIF, LHX2*
Signal transduction	*ALS2, ARL6IP6, DEPDC1, JUB, NOC3L, RAB21, RAB2B, RACGAP1, SLAMF1, SMAD3, STMN1, STRN4*
Transport	*C20orf35, CACNB1, GPD2, MFTC, NUP107, PSCD1, SCN2B, Shax3, SLC19A2, SLC25A17, SLC25A17, THOC4*

**B. Down-regulated genes**	

Apoptosis	*CARD9, DAPK3, DOCK1, GP9, LTBR, YARS, PRKCZ, TNFRSF6B, DDIT4, CLU, HIPK2, NME3, NUDT2*
Cell adhesion	*BCLAF1, CCDC78, CD151, ITGB3BP, LAMA5, LIMS2, MUC6, SN*
Chromatin organization	*H1FX, HIST1H1E, HIST1H2AA, HIST1H3G, HIST1H3H, HMG20B, SAFB*
Cytoskeleton	*ACTR1B, CYLN2, DLC1, DNAI2, EML3, TMEM102, KRT6E, ODF2, PDLIM2, SPTBN5, TUBGCP2, VILL*
Immune & inflam-matory response	*C1QL1, CD2BP2, CIB3, COLEC11, HES6, HLA-B, HLA-C, HLA-E, HLA-G, KIR3DL3, LAT, OAS3, OTUB1, IRGC, RFX1, TNFRSF13B, NIBP*
Lipid metabolism	*ARH, HDLBP, LDLR, LOC197322, PEMT, AGPAT2, CYP11A1, PCCB, PSAP*
Metabolism (other than lipid metabolism)	*AKR1C3, ALDOA, B4GALT2, CA5A, CDC42EP1, CLYBL, DHRS10, ENO1B, GMDS, GRHPR, HAGHL, HPD, HSD11B1, HYAL2, IMPDH2, ITIH2, LARGE, LHPP, NDST2, NUDT14, OGDH, P4HB, PRLH, PSMC3, PYGM, RDH13, ST6GALNAC6, TKT*
Protein biosynthesis & modification	*ASMTL, C9orf54, CRYL1, EEF1A2, EEF1D, EIF4G1, HOXB8, MRPL12, RPL18, RPS14, RPS2, RPS9, SURF6, TUFM, VARS2, PPP2R4, DUSP15, CKAP1, FKBP2, HKE2, PPIB, ST3GAL3, ADCK1, STK11, KIAA1542*
Regulation of transcription	*PRDM9, ANKRD23, ARID5A, ASCL2, CHST7, COMMD4, DUX4, ENO1, EWSR1, FOXQ1, HLA-A, HOXA10, IL17R, ING5, KEAP1, LBX1, LTBP3, MBD3, MED25, MLLT1, MXD4, MYBBP1A, POLR1A, POLR2E, SIAHBP1, SLC6A8, SUPT3H, TEAD2, VGLL4, ZNF511, ZNF607*
RNA processing	*C20orf14, CPSF3L, DDX49, DHX30, EXOSC6, HNRPAB, LOC144983, MARS, PARN, PPAN, RNASET2, SNRPB, ARL6IP4, CIRBP*
Signal transduction	*ADCY2, ADCY5, CABIN1, CALML5, CAMK1, DDEF1, DLL4, EPN1, FZD1, GNA15, GPR37L1, GRAP, GUK1, ITGB1BP2, JAK1, KSR2, MAP2K5, MAPBPIP, MAPKAPK2, NRTN, NSMCE1, OMP, OR2T5, OR2Z1, PIP5K1C, PKIG, PRKCG, PSD, PTPRM, RAP2B, RHO, SPHK1, STOML2, TMEPAI, WNT1*
Transport	*ABCC6, ASNA1, BC-2, CACNA1B, CCS, CORO1A, CYBA, DOT1L, ETFDH, FLJ11749, GRIN2D, HBM, LMAN2, MGC19604, MSCP, NDUFS2, NDUFS6, PP784, SCNN1D, SEC24C, SEC61A1, SEC8L1, SLC12A6, SLC25A29, SLC25A6, SLC2A11, SLC37A1, SNX15, STX10, SYNGR2, TRAPPC6A, GRM4*

**Figure 3:**
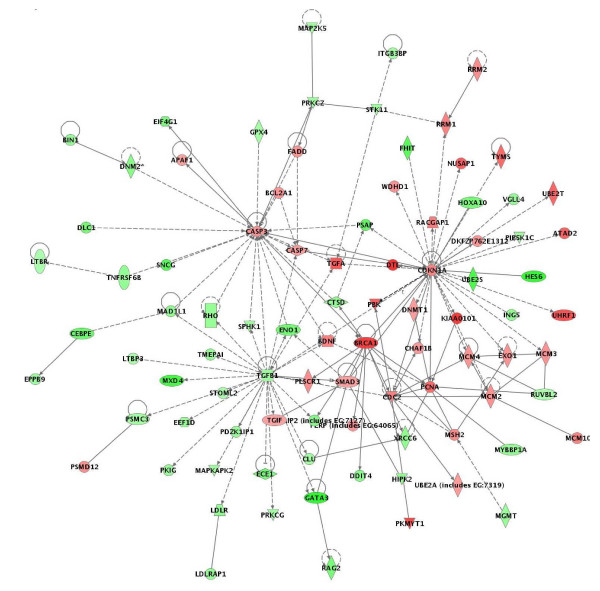
Network analysis of dynamic gene expression in OC spheroids based on the 1.5-fold common gene expression list obtained following cisplatin treatment. The three top-scoring networks were merged and are displayed graphically as nodes (genes/gene products) and edges (the biological relationships between the nodes). Figure legends are as described in Fig. 2.

The above data were further confirmed by pathway and network analyses. Indeed, major functional gene categories that were specifically up-regulated in cisplatin-treated OC spheroids included cellular assembly and organization, cell death and DNA replication, recombination, and repair (Figure 1B). Pathways associated with metabolism, molecular transport, protein synthesis and trafficking were down-regulated, while pathways linked to cell growth and proliferation, cell cycle and cell signaling displayed altered regulation (Figure 1B). Network analysis identified 21 highly significant networks with score ≥ 9 [see Additional file 4]. The 5 top-scoring networks found in cisplatin-treated OC spheroids were associated with cell cycle, cancer, DNA replication, recombination and repair, cancer and altered gene expression (Table 3B). A common network obtained upon merging the three top-scoring networks identified some shared nodes found upon treatment with all drugs (*CDKN1A*, *BRCA1*, *CASP3*, *PCNA*; see above), as well as several specific cisplatin exposure-related nodes implicated in apoptosis and cell cycle control, including cell division cycle 2 (*CDC2*), SMAD, mothers against DPP homolog 3 (*SMAD3*), minichromosome maintenance deficient 2, mitotin (*MCM2*) and transforming growth factor beta 1 (*TGFβ1*) (Figure 3).

#### Topotecan treatment

Three hundred and sixty genes were up-regulated and 663 genes were down-regulated at least 1.5 fold in topotecan-treated OC spheroids [see Additional file 5]. A list of selected functional categories of differentially expressed genes (≥1.5-fold) in topotecan-treated spheroids is shown on Table 5. As seen in Table 5, genes functionally related to apoptosis, cell growth and proliferation, cell cycle control, cell adhesion, cytoskeleton, DNA replication and repair and defense (stress) response, displayed comparative up- and down-regulation upon topotecan treatment. Genes implicated in cell cycle arrest, and protein ubiquitination were predominantly overexpressed, while genes linked to chromatin modification and maintenance, immune and inflammatory response, metabolism (including lipid metabolism), protein biosynthesis and modification, signal transduction and molecular transport, were mostly down-regulated (Table 5).

**Table 5:** Selected differentially expressed gene groups upon topotecan treatment.

**A. Up-regulated genes**	
Apoptosis	*PERP, BAG2, NOL3, PDCD2L, PHLDA2, THAP2, TEGT, CASP3, CASP7, SCOTIN, SMNDC1*
Cell growth and proliferation	*ASPM, CDCA1, DDX11, KIF2C, MAD2L1BP, NEK2, PLOD3, CALM1, CALM2, CDC2, CSRP2, RARRES3, CDCA5, NUSAP1, CTBP2*
Cell cycle	*C14orf11, CCDC5, CCNB2, CDC37L1, CDCA2, CKS2, CNNM4, PKMYT1, SNF1LK, TIMELESS, UBE2C, UHRF1, ZWINT, PLK2, SERTAD1*
Cell cycle arrest	*BTG3, CDKN1A, CDKN2D, HIS1, MFN2, PPM1G, TRIM13, PPM1D*
Cell adhesion	*ANXA9, GJB1, STRN4, TFPI2, TNXB, GJA10, PCDHB16, C3orf4, CLDN1, CLDN2, ANKRD32*
Cytoskeleton	*ANXA9, CFL2, CKAP2, KRT8, SDCBP, SLC9A3R1, SPTAN1, TUBB2, TUBB3, WASF1*
Defense response	*APOBEC3B, CAMLG, UPK3B, ARS2, AHR, HSPA4L, HSPH1, SELS*
DNA replication and repair	*C1orf124, CRY2, DDB2, NEIL1, PCNA, PTTG1, RAD51AP1, TDG, DUT, POLA2, POLG*
Metabolism	*ACOX1, CDS1, ELOVL4, GALGT2, PLA2G4C, PLSCR1, ADIPOR1, GOT1L1, ABHD13, ABHD3, ADHFE1, ARPP-19, GLA, GLS2, GYG2, IDS, PGM2, PGM2L1, PPP1R2, PSMA4, RP2, TEX261, DCK, OBFC2A*
Protein biosynthesis & modification	*ARL6IP, EIF4H, FAM83D, MRPL52, MT-ND3, RPL39, RPLP1, RPS13, RPS29, PPP2CB, PTP4A3, BBS10,, DNAJA5, DNAJB5, DNAJC9, NKTR, PPIC, FUT11, ICMT, KIAA1804, PARP2*
Ubiquitination	*CBLL1, DTX3L, FBXO5, HSPC150, LOC51136, MYLIP, RFFL, RNF139, RNF6, TRIM21, UBE2D1, UBE2C*
Regulation of transcription	*SAFB2, TUB, BACH1, BRD8, C7orf11, CCRN4L, CNOT8, DXYS155E, E2F8, FOXA3, GTF2B, KLF5, LHX2, NR4A1, OSR2, PLAGL2, POLR2A, SAP18, SMYD1, SOX10, SOX9, ST18, SUHW3, SUPT16H, SUZ12, TAF12, YEATS2, ZNF211, ZNF318, ZNF326, ZNF92, DNMT1, EED, GMNN, TCEAL1*
RNA processing	*CSTF2T, DBR1, DDX20, DDX48, FRG1, HEAB, KHSRP, LARP6, PRPF39, RBM26, SF3B4, SLBP, SLU7, ZCCHC8*
Signal transduction	*CABYR, CGA, DKK1, DLG7, DUSP9, DYRK3, FZD4, IER5, MAPK7, NRAS, OR7E91P, PRKAR1A, RAB2B, RACGAP1, RASSF1, RIT2, RKHD3, RUSC1, STMN1, STRN4, TFPI2, TP53TG3, YWHAB*
Transport	*AMPH, APBA3, ATP2B3, CLCC1, COG1, CYP2R1, DYNLT1, GGA3, KCNJ9, KCTD10, KPNA2, LAPTM4A, NDUFB2, NETO2, RBM8A, RINT-1, SCN2B, Shax3, SLC19A2, SLC25A17, SLC25A4, SLC9A6, SMOX, TRAM1, TRAPPC4, TXNL5, WDR48, YIF1*

**B. Down-regulated genes**	

Apoptosis	*APP, CRYAA, DAPK1, DAPK3, DNM2, DOCK1, FAF1, GULP1, NALP12, TNFRSF12A, TNFRSF6B, WDR9, YARS, BCL2L2, FKSG2, PRKCZ, AIFM3, BCLAF1*
Cell adhesion	*ALCAM, CASK, CD151, CDA08, CDH2, CLDN18, EIF4G1, FARP1, GJC1, ITGB5, LAMA3, LAMA5, LAMB1, PKP4, ROBO1, SN, TNR, VCL*
Cell growth & proliferation	*NEK7, CTDSPL, HDGF2, IGF2R, NPDC1, CKLFSF2, AAMP, PRKD1, IL27, NIPSNAP1, PDAP1, PEMT, TPD52L2, ZMYND11, MXD4, IGF1R, LRP16, MT3, MOBKL2B*
Cell cycle	*CCND1, PCTK2, SEPT6, CDK6, CDK8, CHES1, PLK1, STAG1, EXT1, FHIT*
DNA replication and repair	*DDIT4, ECGF1, ERCC8, FBXO18, LIG3, MGMT, PRKDC, POLDIP3, SET*
Chromatin modification & maintenance	*ACTL6B, ANP32B, CHD6, HDAC3, HDAC8, HIST1H1B, HIST1H1E, HIST2H2AC, SAFB, H1FX, HUWE1, SMARCD2, KIAA1797*
Cytoskeleton	*ARPC1A, C9orf140, CENTG2, CEP170, CYLN2, DIAPH2, DLC1, DLG1, DNCH2, FHOD3, FMNL2, GRM4, ITGB1, KIF21A, MGLL, MID1, ODF2, SNCG, SPIRE1, SPTBN1, SPTBN5, SVIL, ARPC4, FMNL1*
Defense response	*CBARA1, DEFQ1, GATA3, GNLY, HSPA12B, NOD27, AHSA1, OXR1, STIP1, STK25, STK3, STK39, TLK1, ZAK, GADD45B*
Immune & inflam-matory response	*AMBP, C1QL1, COLEC11, D2S448, EIF3S8, FN1, HLA-A, HLA-B, HLA-E, IGSF4, IGSF4C, KIR3DL3, MCP, RFX1, RSRC1, TREM1, CCL19*
Lipid metabolism	*PRKAG1, ACAA1, ARH, CHKA, CRYL1, HMGCS1, INPP5E, LDLR, SBF2, SREBF2, HADHA, CRYL1, DHCR24, HDLBP, PIP5K2A, PITPNM1, PSAP*
Metabolism (other than lipid metabolism)	*INSR, GANAB, ABHD14B, ACO2, ADK, ADK, AK5, ALDH4A1, ALDOA, ASMTL, B4GALT2, BCDO2, C10orf110, C3orf26, CA5A, CKB, COG5, CROCC, DHRS10, DHRSX, DHRSX, GBE1, HAGHL, HARS2, HHAT, HIBADH, HS3ST3A1, ITIH2, KIAA0100, LARGE, MAT2A, ME1, ME1, MSRA, MVK, NUDT14, NUDT2, PDHA2, PDSS2, PGAM5, PGD, PHGDHL1, PHKB, PIK4CA, PMM2, PPP1R9A, PSMC3, PYGM, raptor, RDH13, SDHA, ST6GALNAC6, SULF2, SUMF1, ACBD6, TIMM44*
Protein biosynthesis & modification	*ADCY5, EIF2S3, ELP4, LAT, MARS, MRPL12, MRPL12, MRPL23, MRPL28, PARN, RPL10L, RPL22, RPL36, RPL6, RPS14, RPS2, RPS9, RRBP1, SURF6, TUFM, VGLL4, WARS, EEF2, EIF4G3, ATAD3B, BCKDHB, BMPR1A, CDC14B, CKAP1, CYP11A1, DUSP15, FLOT1, IMMP2L, KIAA1542, PAM, PPP2R4, TRIAD3, TRIO, UGCGL2, WNK2, PTPN3, PTPRK, PTPRM, FNTB, CCT7, FKBP8, FKBP9, PPIB, TBCD, ST3GAL4, RFWD2, TTC3*
Proteolysis & peptidolysis	*ADAM9, ADAMTS7, AFG3L2, CAPN5, COH1, CPD, CTSL, DPP7, PRSS15, PRSS8, PSMB7, PSMB9, SERPINB11, SERPINH1, SIPA1L3, SPPL3, TSP50*
Regulation of transcription	*TCF25, ADCY2, ARID1B, ARID3A, ASCL2, ATF4, BAZ2B, BBX, BCL6B, BRF1, CALR, CEBPE, COMMD10, DBP, DEAF1, EEF1A2, EWSR1, FOXQ1, HERC2, HES6, HMGA2, IPF1, ITGB3BP, KLF13, LBX1, MED25, MLLT1, MTA1, POLR2E, PRDM9, PSD, PTK2, SLC2A4RG, SMARCA3, SMARCB1, SND1, SOX4, SPDEF, SUPT3H, TBL1XR1, TCF12, TCF8, TEAD2, TEAD4, TGFB1I4, TRPS1, ZFP3, ZNF768, MLLT3, ZFPM1, ZNF193, ZNF214, ZNF271, ZNF306, ZNF503, ZNF511, ZNF521, ZNF543, ZNF569, MAFK*
RNA processing	*ARL6IP4, CIB3, DDX10, DDX49, DDX56, EIF4B, ENO1B, EXOSC6, HNRPA2B1, HNRPAB, HNRPUL1, KARS, LOC144983, NARS2, PABPC4, QKI, RALY, RNASET2, RNGTT, RPP40, SFRS5, SNRPB, SNRPN*
Signal transduction	*PPARGC1B, AIG1, AKT1, BRE, C16orf45, C20orf23, CABIN1, CAMK1, CHST7, CSNK1A1, CSNK1A1L, DDEF1, EDG6, EPS15, FZD1, GNAI1, GNAQ, GNB1, GNB2L1, GNG8, GPR172B, GPS1, GRAP, GRK1, HCG9, IL17R, INADL, INPP4B, ITGB1BP2, ITM2C, JAK1, KIAA0174, KRT6E, LMBR1, LPP, MAP2K2, MAP3K4, MAP4K3, MAP4K5, MAPBPIP, MRAS, MYH13, NMBR, NRTN, OMP, OR2T5, OR2Z1, PITPNC1, PKIG, PRKCSH, PRR14, PSTPIP1, PTD004, PTK7, PTK9L, PTPN11, RAB31, RAC3, RAN, RAP2B, RHO, SMAD3, SPHK1, STC2, STOML2, TNFRSF13B, TRABD, TRIM54, USH1C, WNT1, WNT3A, LPHN2*
Transport	*ABCC1, ABCC4, ABCC6, ACTR1B, ANXA6, AP1S3, ATP5G2, ATP6V0E2, CHMP6, CLIC4, COL4A1, CORO1A, COX7A2L, CYC1, DLG2, DOT1L, EPN1, ETFDH, FLJ10815, FLJ22659, FTHL17, FTL, GABARAP, HBM, ITSN1, KCNJ13, KCNK3, KDELR1, KPNA4, LMAN2, LYRM4, MSCP, NBEA, NDUFA12L, NDUFS2, NDUFS6, NDUFS7, NQO2, PTPRG, R30953_1, RAB1A, RAG2, SCNN1D, SEC14L5, SEC24C, SEC5L1, SEC61A1, SEC8L1, SFXN4, SLC12A6, SLC25A29, SLC37A1, SLC6A8, SNX15, SNX17, SYNE2, THADA, TUSC3, TXN2, TXNDC4, TXNRD1, SLC25A6, FCHSD2*

**Figure 4:**
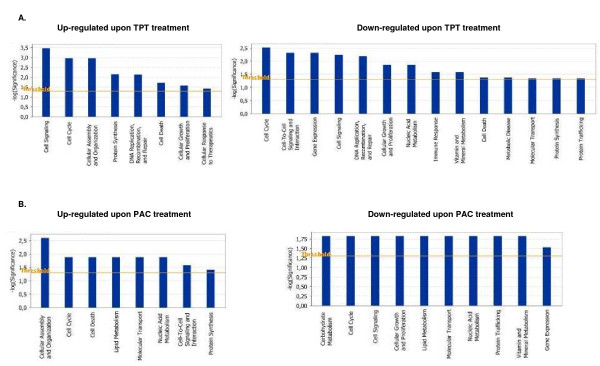
Functional analysis for a dataset of differentially expressed genes (≥1.5 fold) in OC spheroids following CT drugs treatments. A. Functional analysis following topotecan treatment, B. Functional analysis following paclitaxel treatment. Top functions that meet a *p*-value cutoff of 0.05 are displayed.

**Figure 5:**
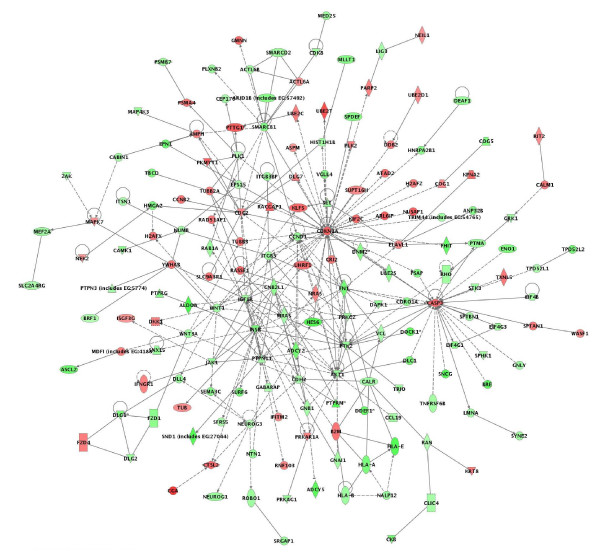
Network analysis of dynamic gene expression in OC spheroids based on the 1.5-fold common gene expression list obtained following topotecan treatment. The five top-scoring networks were merged and are displayed graphically as nodes (genes/gene products) and edges (the biological relationships between the nodes). Figure legends are as described in Fig. 2.

IPA validation of biological functions and networks that were most significant to the topotecan microarray data set were in agreement with our initial gene expression data. As shown on Figure 4A, functional pathways implicated in cell growth and proliferation, cell cycle, cell death, cell signaling, DNA replication, recombination and repair and protein synthesis displayed significant altered expression in both directions. Positively induced pathways comprised those linked to cellular assembly and organization and cellular response to therapeutics, while functional pathways that were subject to down-regulation in topotecan-treated OC spheroids were associated with cell-to-cell signaling and interaction, metabolism, immune response, protein trafficking and molecular transport (Figure 4A). Thirty highly significant networks with score ≥ 9 were identified by network analysis [see Additional file 6]. The five top-scoring networks were functionally associated with DNA replication, recombination, and repair, cellular assembly and organization, cell cycle, cellular movement, cell death, protein trafficking and molecular transport (Table 3C). A common network was obtained upon merging the five top-scoring topotecan-related networks (Figure 5), which recognized some nodes found also in the all-drugs-treatment networks (*CDKN1A*, *CASP3 *(up-regulated), and *CCND1*, *SMARCB1*, *INSR*, *PTK2*, *HLA-A*, *PTPN1*1 (down-regulated) and in the cisplatin-treatment network (CDC2). Additionally, the network analysis identified specific topotecan-related down-regulated gene nodes that include v-akt murine thymoma viral oncogene homolog 1 (*AKT1*), protein tyrosine kinase 2 (*PTK2*), wingless-type MMTV integration site family, member 1 (*WNT1*), insulin-like growth factor 1 receptor (*IGF1R*), fibronectin 1 (*FN1*) and calreticulin (*CALR*). These nodes comprise genes, mainly associated with cell cycle progression and cell proliferation (*AKT1*, *WNT1*, *IGF1R*), cell adhesion (*FN1*, *CALR*) and cell invasion (*PTK2*).

#### Paclitaxel treatment

**Figure 6:**
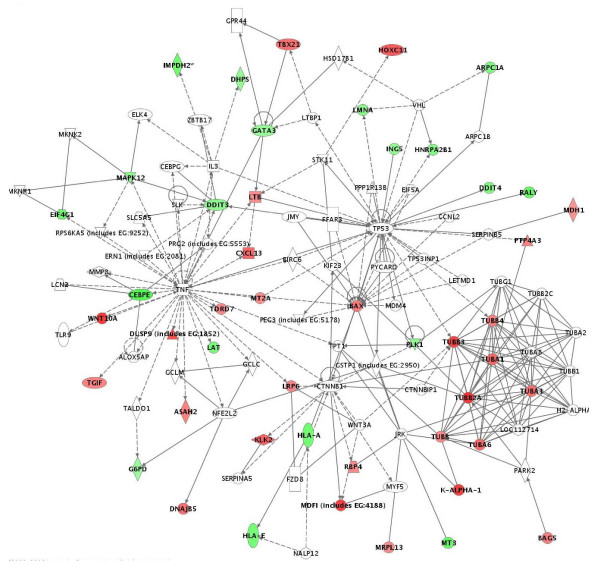
Network analysis of dynamic gene expression in OC spheroids based on the 1.5-fold common gene expression list obtained following paclitaxel treatment. The three top-scoring networks were merged and are displayed graphically as nodes (genes/gene products) and edges (the biological relationships between the nodes). Figure legends are as described in Fig. 2.

We found 126 genes to be up-regulated and 139 genes to be down-regulated at least 1.5 fold in paclitaxel-treated OC spheroids [see Additional file 7]. Table 6 shows list of selected functional groups of these genes. Thus, up-regulated genes upon paclitaxel exposure are implicated in apoptosis, cell adhesion and cytoskeleton structure (including a number of tubulin genes), while down-regulated functional groups comprised genes linked to cell growth and proliferation, immune response and transcription regulation. Comparatively high number of genes with similar function displayed proportional up- and down-regulation upon paclitaxel treatment, and more specifically, genes related to cell cycle control, metabolism, protein biosynthesis and modification, signal transduction and transport. Network analysis identified up-regulated functional pathways linked to cellular assembly and organization, cell death and protein synthesis, while down-regulated pathways included cellular growth and proliferation, control of gene expression, and protein trafficking. Pathways, associated with cell cycle, metabolism, transport and cell signaling displayed comparative altered expression in both directions (Figure 4B). Eleven significant networks were identified following paclitaxel exposure [see Additional file 8], and the five top-scoring pathways were mostly associated with cancer, cell death, drug metabolism, gene expression, molecular transport and inflammatory disease (Table 3D). A common network obtained upon merging the three top-scoring networks identified the pro-apoptotic BCL2-associated X protein (*BAX*) node and several tubulin genes, that were up-regulated upon paclitaxel treatment, as well as a number of differentially expressed genes linked with the p53 and the tumor necrosis factor (*TNF*) pathways (Figure 6).

**Table 6:** Selected differentially expressed gene groups upon paclitaxel treatment.

**A. Up-regulated genes**	
Apoptosis	*BAG5, GULP1, BAX*
Cell cycle	*SEPT10, C10orf9, TAF1L*
Cell adhesion	*CELSR3, CLDN2, COL24A1, SYMPK, TNXB, CTTN, GJA10*
Cytoskeleton	*CDC42, K-ALPHA-1, TUBA1, TUBA3, TUBA4, TUBA6, TUBB, TUBB2, TUBB3, TUBB4, TUBB6*
Metabolism	*EBPL, OSBP, OSBP2, PAQR6, AMAC1L2, AOC2, ASAH2, CL640, MDH1, NDUFA4, FAM3D*
Protein biosynthesis & modification	*MRPL13, RPL39, RPS29, PTP4A3, DNAJB5, MGAT2, RNF149, RNF44, TRIM21*
Response to stress	*MT2A, C2D1A, IKIP, TFG*
Signal transduction	*BZRP, DUSP9, EFNA3, LPHN1, LRP6, NRAS, SNX24, STARD8, WNT10A*
Transport	*ATP2B3, CNO, GRASP, KCNJ9, PRRG2, RBP4, S100A10, SLC25A17, SLC25A24, TMCO3, TPCN2, TRAM2*

**B. Down-regulated genes**	

Cell growth & proliferation	*HDGF2, PDAP1, MT3, DOT1L, H2BFS, HIST1H1B, SAFB, VCX-C*
Cell cycle	*PARD6G, PLK1, DDIT3, DDIT4, MXD4*
Immune response	*C1QL1, HLA-A, HLA-E, LAT, SLPI, TNFRSF13B*
Metabolism	*HDLBP, PSAP, ALDOA, DHPS, DHRS10, ENO1B, G6PD, HAGHL, IMPDH2, NUDT14, PSMC3, SDHA*
Protein biosynthesis & modification	*EIF4G1, MRPL12, MRPL23, RPL10L, VARS2, FLOT1, BCKDK, CKAP1, GALNT9, KIAA1542*
Regulation of transcription	*ZNF317, ZNF225, ATF5, CEBPE, DBP, ZNF665, ZFP3, ZFP41, ING5, MAF1, MBD3, NFE2L1, POLR2E, ZBTB16, ZFP276, ZNF208, ZNF214, ZNF221, ZNF257, ZNF425, ZNF432, ZNF473, ZNF511, ZNF529, ZNF583, ZNF585B, ZNF79, ZNF155*
Signal transduction	*DUSP24, ARHGEF10L, GPR172B, GPR20, GRM4, MAPK12, OR2T5, PRKCSH, RAB3D, TMEPAI*
Transport	*CLTB, COX6A2, EPN1, GRPEL1, KCNJ13, SEC14L5, MGC19604, RNP24, SLC12A6, SLC37A1, TIMM44, TLOC1, ATP5B, ABCC6*
Response to stress	*MT3, GATA3, HSPA1A*

### Association of gene expression patterns with spheroid's morphology

**Figure 7:**
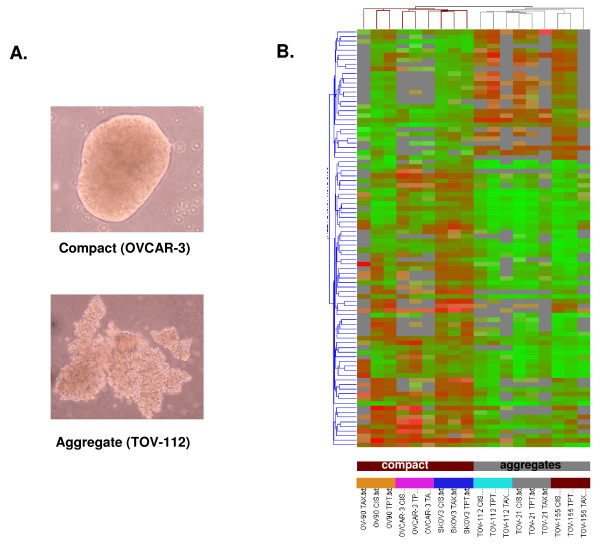
A. Example images of compact and aggregate spheroid structures derived from OC cells. B. Hierarchical clustering of OC spheroids following treatment with all used drugs (cisplatin, topotecan and paclitaxel (taxol)), that discriminates between compact spheroids and aggregates. A subset of candidate genes were initially obtained by filtering on signal intensity (2-fold), retaining 527 genes. One-way ANOVA parametric test (Welch *t*-test, variances not assumed equal, *p *≤ 0.03) further selected 85 genes. Clustering analysis based on the 85 gene list was performed using the standard Condition Tree algorithm provided in GeneSpring. The mean appears *grey*, whereas *red *signifies up-regulation, and *green *signifies down-regulation (see legend bar). Compact spheroids are indicated in *brown*, aggregates are indicated in *grey*. Each cell line is indicated with different color.

The six OC cell lines used in this study displayed different morphology when grown as spheroids, forming rather compact spheroids (derived from OV-90, OVCAR-3, SKOV-3), or more loose structures or aggregates (derived from TOV-112, TOV-21, TOV-155; examples for both spheroid structures are shown on Figure 7A). As expected, a higher number of genes displayed differential expression upon CT drugs treatment in the aggregates than in the compact spheroids (data not shown). These structure-associated gene expression differences were further confirmed by cluster analysis. Indeed, supervised clustering based on a selected list of 85 genes revealed formation of two major cluster groups that perfectly distinguish between compact and aggregate structures (Figure 7B). Genes implicated in cell adhesion (*CSPG3*, *ITGAV*, *MUC1*), negative regulation of cell proliferation (*GPNMB*, *MXD4*) and metabolism (*FGF14*, *CDC42EP1*, *IGFBP4*, *GNA15*, *ARL7*, *HRMT1L1*, *LRDD*, *OR2A1*) were comparatively up-regulated in the compact spheroids, while genes associated with cell proliferation (*ERBB4*, *ADRA1B*, *BMP6*), inflammation (*SERPING1*, *CXCL9*) and protein modification (*DUSP21*, *FLJ23356*, *HSPA1A*) were predominantly up-regulated in aggregates [see Additional file 9]. Each cell line displayed a separate gene cluster regardless of the drug used (Figure 7B), while no significant clusters were obtained for each specific drug treatment (data not shown). Interestingly, cell lines displaying quite different responses to cytotoxics when grown as monolayers (for example SKOV-3 and OVCAR-3) now show very similar cluster patterns upon treatment as they cluster adjacent to each other (Figure 7B), indicating that differences in drugs response tend to disappear when these OC cell lines are grown as multicellular spheroids.

### Validation of microarray findings with semi-quantitative RT-PCR (sqRT-PCR)

To validate microarray results, we arbitrarily selected 16 differentially expressed genes following different drugs treatments and quantified their expression by sqRT-PCR in control and treated spheroids. All sqRT-PCR analyses were performed in samples previously used for the microarray experiments. Table 7 summarizes the gene expression measurements of all validated genes. We found that both methods (microarray analysis and sqRT-PCR) detected similar patterns for the up- and down-regulated genes selected for validation.

**Table 7:** Semi-quantitative RT-PCR validation of microarray data.

**Treatment**	**Gene**	**Common name**	**Fold expression**		**p-value**
			
			**Microarray**	**sqRT-PCR**	
All	BRCA1	breast cancer 1	2,12	1,44	0,0257
All	*CASP7*	caspase 7	1,53	1,48	0,0625
All	*COL25A1*	collagen, type XXV, alpha	3,83	1,48	0,0313
All	*TLR8*	toll-like receptor 8	3,6	1,55	0,0049
All	*COX2*	cytochrome c oxidase II	1,57	1,31	0,0562
All	*SEC8*	secretory protein SEC8	-2,02	-1,27	0,0488
cisplatin	*GMNN*	Geminin	1,97	1,39	0,0612
cisplatin	*KCNC1*	potassium voltage-gated channel, 1	3,56	1,26	0,0313
cisplatin	*LY96*	Lymphocyte antigen 96	9,96	1,78	0,0303
cisplatin	*TYMS*	thymidilate synthase	2,25	1,3	0,0244
topotecan	*AHR*	aryl hydrocarbon receptor	1,98	1,43	0,0386
topotecan	*NR4A1*	nuclear receptor subfamily 4, A1	3,17	1,4	0,033
topotecan	*S*100*A*2	S100 calcium binding protein A2	2,27	1,72	0,0247
topotecan	*HLA-E*	major histocompatibility complex, IE	-3,02	-1,25	0,0231
paclitaxel	*TUBA4*	tubulin, alpha	2,59	1,35	0,0247
paclitaxel	*TUBB3*	tubulin, beta 3	2,51	1,62	0,0135

## Discussion

In attempt to identify detailed molecular mechanisms of drugs actions and early response to CT treatment, we performed gene expression profiling of OC spheroid cultures treated with cisplatin, topotecan and paclitaxel. To our knowledge, the present work represents the first effort to define global changes in gene expression in CT drugs-treated OC spheroid models by using high-density microarrays. We used six OC cell lines for spheroid formation that displayed diverse response following treatment with different drugs in order to identify common mechanisms of early response to drug action in OC. Indeed, the six monolayer cell cultures displayed variable chemosensitivity against cisplatin, while following paclitaxel and topotecan treatment, the IC50 values were very similar for all cell lines with one exception: the TOV-21 line showed significantly higher IC50 value upon topotecan treatment than the remaining cell lines (Table 1). However, these differences tend to disappear as the same cell lines were grown as multicellular spheroids. We have checked for the numbers of apoptotic cells in paraffin embedded spheroid structures by DAPI staining and were not able to find significant differences in drugs-treated spheroids with similar morphology (compact spheroids or loose aggregates) that were propagated from cell lines displaying quite variable chemosensitivities as monolayers (data not shown). The above observation was also confirmed by our cluster analysis.

We analyzed both functionally related genes that were commonly differentially expressed following all drugs treatments, as well as alterations in gene expression patterns that were specific for each of the three drugs used (cisplatin, topotecan and paclitaxel). As the main goal of CT treatment is the induction of apoptosis, we noticed a comparative number of apoptosis-related genes that were up- and down-regulated following all treatments (Table 2). Similar expression patterns of apoptosis-related genes were observed upon cisplatin and topotecan treatments, while paclitaxel uniquely induced the up-regulation of several pro-apoptotic genes (*BAG5*, *GULP1 *and *BAX*). While induction of pro-apoptotic genes is expected upon treatment with chemotherapeutics, the down-regulation of genes implicated in apoptosis upon exposure to cisplatin and topotecan could be indicative for some compensatory mechanisms, linked to drugs-induced cell death [20].

Treating spheroids with chemotherapeutics affected strongly the regulation of the cell cycle, as a number of genes implicated in cell cycle control, including genes involved in cell cycle arrest, were found to be induced especially following cisplatin and topotecan treatments. Although cell cycle deregulation events could lead to apoptosis induction, cell cycle arrest could constitute a significant event in the survival of the spheroids following a major stress like CT treatment [21,22]. Moreover, cell cycle arrest is essential for the activation of DNA repair mechanisms [23], as a number of DNA repair genes (*BRCA1*, *BRCA2*, *DDB2*, *FANCA*) were found to be up-regulated in OC spheroids following all treatments. Indeed, *BRCA1 *and *FANCA *(a member of the Fanconi Anemia family of proteins) are involved in detecting DNA damage, causing cell cycle arrest and allowing DNA repair, which contributes to chemoresistance [24], while disruption of the *BRCA1 *pathway may promote sensitivity to platinum-based therapies [25,26]. A marked up-regulation of genes encoding for DNA replication and repair proteins was particularly evident following exposure to cisplatin (Table 4A). This was not surprising since cisplatin action in OC cells implies the formation of DNA adducts that enable physiological processes such as replication and transcription, leading to oxidative stress and enhanced DNA repair [27,28]. Topotecan treatment led to comparative up- and down-regulation of DNA replication and repair genes (Table 5). Thus, our data obtained with the OC spheroid model are confirmative to previously proposed mechanisms of cisplatin and topotecan action in OC cells associated with enhanced DNA replication and repair [29,30].

Likewise, different genes functionally involved in cellular assembly and organization (including cell adhesion and cytoskeleton organization) were comparatively induced or suppressed following exposure to the three drugs used. Indeed, a number of cell adhesion-related genes, including some integrins, cadherins and claudins, were down regulated upon all drugs treatments (Table 2B). As demonstrated, a destabilization of the cellular adhesion in spheroids following treatment was causal for apoptosis induction [31]. Moreover, it is recognized that the stability of the cellular adhesion in spheroid cultures is closely related to the stability of the cell cytoskeleton [32]. Consequently, various genes functionally implicated in cytoskeleton organization were commonly down-regulated after all treatments (Table 2B). However, a common overexpression of cell adhesion and cytoskeleton-related genes was also observed in all treated OC spheroids, as well as upon exposure to cisplatin or topotecan, which probably represents an adaptive answer to CT treatment (Tables 2A, 4A and 5A).

Paclitaxel treatment resulted exclusively in the induction of cell adhesion- and cytoskeleton-related genes, (including numerous tubulin genes) in OC spheroids (Table 6A). Although class III beta-tubulin overexpression was previously associated with paclitaxel resistance in OC [33], to our knowledge, such a major overexpression of various tubulin genes in cancer cells upon paclitaxel exposure has been not demonstrated previously. Since the principal mechanism of paclitaxel action implies a deregulation of the microtubules dynamics by promoting and stabilizing microtubule formation and inhibiting microtubule depolymerization [34], the increased expression of different tubulin isotypes could partially compensate paclitaxel action by altering the dynamic equilibrium between soluble and polymerized tubulin that exists in the absence of drug.

Our data support previous reports that multicellular/adhesion-mediated survival mechanism could render both normal and cancer epithelial cells less vulnerable to undergoing drug-induced apoptosis [35,36]. Indeed, multicellular resistance can only be demonstrated in three-dimensional cultures and fails to be shown in monolayers or cell suspensions. This is explained by the fact that cell-to-cell and cell-to-stroma adhesion limits drug penetration, and by the variable susceptibility to cytotoxicity determined by oxygen and tissue proliferation gradients [17]. Thus, an increase of cellular adhesion in spheroids was previously associated with a resistance to antineoplasic agents [17,37]. Our comparison of the gene expression patterns in OC spheroids with different morphology, including compact spheroids or more loose structures (cellular aggregates), demonstrated certain differences in response to treatment, as found by gene cluster analysis (Figure 7B). More specifically, gene clustering was indicative for overexpression of several cell adhesion genes (*CSPG3*, *ITGAV*, *MUC1*) in the compact spheroid structures in comparison to the aggregates, which could be determinative for the observed variations in spheroid's morphology and response to treatment. Taken together, these results suggest that some aspects of cisplatin, topotecan, or paclitaxel early resistance in ovarian carcinoma may be multicellular/adhesion-dependent or associated [38-40].

As expected, upon all drugs treatments we monitored down-regulation of numerous genes that are functionally associated with metabolism, signal transduction, gene expression, protein biosynthesis and modification, immune response/inflammation, and molecular transport. As a matter of fact, suppressed transcription and lower metabolism rates have been previously linked with chemotherapeutics action [41-43]. We want to specifically note that a significant number of genes involved in lipid metabolism were found to be suppressed in CT drugs-treated OC spheroids (Table 2B). Indeed, OC patients exhibit altered lipid metabolism and the degree of these alterations have been previously linked with response to therapy, as these metabolic alterations may influence disease outcome [44,45]. Our previous work suggested that lower lipid metabolism rates might improve treatment response in OC patients [13]. Thus, the observed lower expression of genes involved in lipid metabolism might be associated with an immediate mechanism of drugs action in OC spheroids.

Although identification of a list of individual genes that show expression changes is important, there is an increasing need to move beyond this level of analysis. Instead of simply enumerating a list of genes, we wanted to know how they interact as parts of complexes, pathways and biological networks. For this purpose, the microarray data were imported into the IPA software to identify relevant biological pathways and networks. Pathway and network analyses were highly confirmative of the gene expression data obtained. Indeed, specific functional pathways displayed similar expression patterns in cisplatin- or topotecan-treated OC spheroids which were rather comparable to those observed upon all drugs treatments (Figures 1A,1B and 2A). Network analysis was indicative for important roles of some genes and related networks (*BRCA1*, *CDKN1 *(*p21*) and *CASP3*) in the mode of action of these drugs (Figures 3, 4, 5). This similarity was not unexpected because both cisplatin and topotecan action triggers the formation of DNA lesions which interfere with and inhibit DNA replication.

Network analysis was also suggestive for the important role of *TGFβ1 *and related network in cisplatin action (Figure 4). The role of *TGFβ1 *in the cellular response to cisplatin is not well defined. However, *TGFβ1 *is known to be involved in tumor suppression [46,47] and its down-regulation following cisplatin treatment could represent a compensatory mechanism to this drug action.

Topotecan, a topoisomerase-I inhibitor, is most frequently used as a second-line CT treatment of recurrent OC [43]. Analysis of the various biologic pathways and networks implicated in the cellular response to topotecan showed similarities with cisplatin action (including over-expression of *CASP3*, *CDKN1*, *CDC2*; see Figure 5). However, various other genes and related pathways, associated with cell growth and survival (*AKT1*, *WNT1*, *IGFR1*, *CCND1*) were specifically down-regulated after topotecan treatment (Figure 5), which is probably relevant to the specific mechanisms of cytotoxic action of this drug in OC cells.

Pathway and network analyses of paclitaxel action in OC spheroids demonstrated comparative up- and down regulation of genes functionally associated with metabolism, protein biosynthesis and modification, signal transduction and transport, and were again quite confirmative of the gene expression data obtained with this drug (see Figures 2A and 6).

## Conclusion

We used the OC spheroid model to define global changes in gene expression that are linked to the molecular mechanisms of CT drugs action and early response to treatment. Exposure of OC spheroids to significantly high concentrations of CT drugs such as cisplatin, topotecan and paclitaxel (see Table 1) resulted in differential expression of genes, functionally associated with to cell growth and proliferation, cellular assembly and organization, cell death, cell cycle control and cell signaling. Genes and corresponding pathways implicated in cell cycle arrest, DNA replication and repair were predominantly overexpressed, while genes associated with transcription control, metabolism, transport, immune and inflammatory response were mostly down-regulated. Cisplatin and topotecan treatments triggered similar alterations in gene and pathway expression patterns, while paclitaxel action was mainly associated with induction of genes and pathways linked to cellular assembly and organization, cell death and protein synthesis. Most alterations in gene expression were directly related to mechanisms of cytotoxic actions of the CT drugs in OC spheroids. However, the induction of genes linked to mechanisms of DNA replication and repair in cisplatin- and topotecan-treated OC spheroids could be associated with immediate compensatory response to treatment. Similarly, overexpression of different tubulin genes upon exposure to paclitaxel could represent an early adaptive effect to this drug action. Finally, changes in relevant drug-resistance associated gene expression brought about by multicellular growth conditions that are known to alter gene expression (including cell adhesion and cytoskeleton organization), could substantially contribute in reducing the initial effectiveness of CT drugs in OC.

Results described in this study underscore the potential of the microarray technology for unraveling the complex mechanisms of CT drugs actions in OC spheroids and initial protective responses to cytotoxic treatment. Although not directly clinically relevant, our data could be indicative for some early events that might be implicated in the onset of acquired OC chemoresistance.

## Methods

### Cell cultures and spheroid formation

The OC cell lines used were either purchased from ATCC (OVCAR3, SKOV3), or were previously isolated and characterized (TOV-112, TOV-21, OV-90), as described [48]. The TOV-155 cell line was recently established in our lab as spontaneously immortalized cyst adenoma cell line and the detailed characterization of this cell line will be reported elsewhere. More detailed information of the cell lines used is presented on Table 1. Multicellular spheroids were prepared by a liquid overlay method [49]. Briefly, the wells were coated with 0.5 ml of 0.6% agarose containing serum-free medium. Tumor cells grown in the complete medium were then transferred to the top of solidified agarose. After culturing for 3 days, multicellular spheroids were formed on the agarose surface. The culture medium was changed partially every 24 h.

### Gene expression profiling and data analysis

Gene expression analysis was carried out as previously described [13,14]. Briefly, total RNA was isolated from CT drug-treated or control OC spheroid cultures using the Trizol reagent (Invitrogen, Burlington ON, Canada). The quality of all RNA samples was examined by capillary electrophoresis using the Agilent 2100 Bioanalyzer (Agilent, Palo Alto, CA). Fluorescently labeled cDNAs were generated from 20 μg of total RNA in each reaction using the Agilent Fluorescent Direct Label Kit and 1.0 mM Cyanine 3- or 5-labeled dCTP (PerkinElmer, Boston, MA), and following user's manual. Cyanine-labeled cDNA from CT drug-treated spheroids was mixed with the same amount of reverse-color cyanine labeled cDNA from the corresponding control (vehicle-treated) spheroids and were applied to the Human 1A (v2) Oligonucleotide Microarray (Agilent), containing 20,174 genes. Upon hybridization and washing, the arrays were scanned using a dual-laser DNA microarray scanner (Agilent). The data were then extracted from images by the Feature Extraction software 6.1 (Agilent). The GeneSpring software (Agilent) was used to generate lists of selected genes and for different statistical and visualization methods. An Intensity-Dependent Normalization (known as Lowess normalization) was applied to correct for artifacts caused by nonlinear rates of dye incorporation as well as inconsistencies of the relative fluorescence intensity between some red and green dyes. Consecutive lists of differentially expressed genes were generated considering a 1.5-fold expression as gene selection criteria for the different experimental conditions are indicated in the Results section. Comparisons of gene expression in compact spheroids and loose aggregates were performed by Cluster Analysis the using Condition Tree algorithm. The genes in the gene lists were classified according to their function using the Gene Ontology (GO SLIMS) classification system. Network analysis of the microarray data was completed using the Ingenuity Pathway Analysis software [50].

### Semi-quantitative duplex RT-PCR (sqRT-PCR)

Validation of microarray data was performed for selected differentially expressed genes by sqRT-PCR as previously described [13,14]. The peptidylprolyl isomerase A-like (*PPIAL*) gene was uniformly expressed in all OC spheroid cultures analyzed (control and treated) and was used as internal standard. Primers were designed for these loci with the sequences freely available from the Entrez Nucleotide database and the Primer3 algorithm for primer design [51]. One-fifth of each PCR reaction was run on a 1.5–2% agarose gel in 1×TBE buffer (45 mM Tris/borate/1 mM EDTA); the gel was documented using the AlphaImager 2200 gel documentation system (Alpha Innotech, San Leandro, CA) and analyzed using the publicly available NIH ImageJ 1.33u program [52]. Each expression value was calculated as a relative ratio between the signal of the specific PCR fragment and that of the internal *PPIAL *standard. The data obtained were statistically analyzed by the paired *t*-test using the GraphPad InStat Software version 3.06 (San Diego CA).

### MTT assay

The MTT cell proliferation assay (Sigma, St-Louis, MS, USA) was used to measure the cell growth inhibition effects of the CT drugs (cisplatin, topotecan and paclitaxel) in the six OC cell lines, grown as monolayers. Cell suspensions (at 2 × 10^4 ^cells/ml) were transferred to 96-well plates in multiple replicates (×5) and incubated for 3 days with different drug's concentrations (ranging between 1 nM and 100 μM). Then, 38 μl of 3-[4,5-dimethylthiazol-2-yl]-2,5-diphenyl-tetrazolium bromide (MTT, 5 mg/ml) was added to each well 4 h before the end of the incubation. After centrifugation and removing the supernatant, 200 μL of dimethyl sulphoxide (DMSO) were added to resolve the crystals and the optical density was measured by microplate reader at 595 nm.

## Authors' contributions

SL'E performed the spheroid and cell line treatment experiments, the microarray experiments and consecutive data validation, and assisted with the discussion. MB assisted in cell culture, spheroid RNA preparation and its qualitative and quantitative analysis and helped for the microarray experiments. BT helped with the establishment and the characterization of one of the OC cell lines used (TOV155) and helped draft the paper. AM-M-M supplied most of the OC cell lines, assisted in the design of the study and helped draft the paper. DB was the Principal Investigator, instigated and designed the study, and helped draft the paper. All authors have read and approved the final version of the manuscript.

## Supplementary Material

Additional file 1 Supplemental Table 1. List of common differentially expressed genes (≥1.5-fold) in cisplatin-, topotecan- and paclitaxel-treated OC spheroids Click here for file

Additional file 2 Supplemental Table 2. Networks with significant score, commonly affected following cisplatin, topotecan and paclitaxel treatments in OC spheroids Click here for file

Additional file 3 Supplemental Table 3. List of differentially expressed genes (≥1.5-fold) in cisplatin-treated OC spheroids Click here for file

Additional file 4 Supplemental Table 4. Networks with significant score, affected by cisplatin treatment in OC spheroids Click here for file

Additional file 5 Supplemental Table 5. List of differentially expressed genes (≥1.5-fold) in topotecan-treated OC spheroids Click here for file

Additional file 6 Supplemental Table 6. Networks with significant score, affected by topotecan treatment in OC spheroids Click here for file

Additional file 7 Supplemental Table 7. List of differentially expressed genes (≥1.5-fold) in paclitaxel-treated OC spheroids Click here for file

Additional file 8 Supplemental Table 8. Networks with significant score, affected by paclitaxel treatment in OC spheroids Click here for file

Additional file 9 Supplemental Table 9. Genes, identified by clustering (the 85 gene list) that are up- or down-regulated in compact OC spheroids in comparison with OC aggregates (≥2-fold, p = 0.03) Click here for file
